# Perceptions on a mobile health intervention to improve maternal child health for Syrian refugees in Turkey: Opportunities and challenges for end-user acceptability

**DOI:** 10.3389/fpubh.2022.1025675

**Published:** 2022-11-22

**Authors:** Christina L. Meyer, Aral Surmeli, Caitlyn Hoeflin Hana, Nirmala P. Narla

**Affiliations:** ^1^Center for Global Noncommunicable Diseases, RTI International, Seattle, WA, United States; ^2^HERA Inc, Boston, MA, United States; ^3^Harris School of Public Policy, University of Chicago, Chicago, IL, United States

**Keywords:** mobile health (mHealth), refugees, digital health, maternal child health (MCH), user experience (UX)

## Abstract

**Background:**

Mobile health (mhealth) technology presents an opportunity to address many unique challenges refugee populations face when accessing healthcare. A robust body of evidence supports the use of mobile phone-based reminder platforms to increase timely and comprehensive access to health services. Yet, there is a dearth of research in their development for displaced populations, as well as refugee perspectives in design processes to improve effective adoptions of mhealth interventions.

**Objective:**

This study aimed to explore healthcare barriers faced by Syrian refugee women in Turkey, and their perceptions of a maternal-child health mobile application designed to provide antenatal care and vaccine services. These findings guided development of a framework for enhancing acceptability of mobile health applications specific to refugee end-users.

**Methods:**

Syrian refugee women who were pregnant or had at least one child under the age of 2 years old at the time of recruitment (*n* = 14) participated in semi-structured in-depth interviews. Participants had the opportunity to directly interact with an operational maternal-child health mobile application during the interview. Using a grounded theory approach, we identified critical factors and qualities mhealth developers should consider when developing user-friendly applications for refugees.

**Results:**

It was observed that a refugee's perception of the mobile health application's usability was heavily influenced by past healthcare experiences and the contextual challenges they face while accessing healthcare. The in-depth interviews with refugee end-users identified that data security, offline capability, clear-user directions, and data retrievability were critical qualities to build into mobile health applications. Among the features included in the maternal-child health application, participants most valued the childhood vaccination reminder and health information features. Furthermore, the application's multi-lingual modes (Arabic, Turkish, and English) strengthened the application's usability among Syrian refugee populations living in Turkey.

**Conclusions:**

The inclusion of refugee perceptions in mhealth applications offers unique developer insights for building more inclusive and effective tools for vulnerable populations. Basic upfront discussions of the mobile application's health goals and its personal value to the user may improve their long-term use. Further prospective research is needed on retention and use of mobile health applications for refugee women and other displaced populations.

## Introduction

Since the start of the Syrian Civil War in 2011, 12 million Syrians have been forcibly displaced from their homes. Turkey hosts nearly four million of these refugees, which constitutes the largest population of refugees in the world (1). Healthcare services are free in Turkey for the Syrian refugee population through national insurance. However, despite these services, there has been an increase in morbidity and mortality among Syrian refugees, particularly women and children (2). Outbreaks of vaccine-preventable diseases among Syrian refugee children in Turkey are increasing despite numerous immunization campaigns (3). Similarly, pregnant Syrian women appear particularly susceptible to the ill effects of missing preventive prenatal and antenatal care. Peer-reviewed studies in Turkish hospitals indicate that refugee women are more likely to die during and after labor (2) and fewer than 50% of pregnant Syrians attend at least four prenatal care visits (4).

A robust body of research evidence supports the use of mobile phone-based reminder platforms to increase timely and complete access to health services, such as childhood immunizations and prenatal care (5–7). A systematic review found that the majority of text message reminder interventions improved health outcomes (8). Additionally, a multinational review of Syrian refugees and other vulnerable Arab populations indicated that these populations have widespread access to mobile phones and cellular networks (9). While specific Syrian refugee data in Turkey is difficult to obtain beyond market surveys, in a recent study with over 1,000 participants, 95.5% of Palestinian refugees in Jordan reported having a mobile phone (10). Additional qualitative studies demonstrate that mobile health interventions may be acceptable and feasible for refugee populations (9, 11, 12).

With numerous studies showing access to technology for refugee populations, efforts to develop useful maternal-child health applications are currently underway. For example, UNRWA, the United Nations Relief and Works Agency created for Palestinian refugees, has deployed a mobile health application that shares maternal-child health educational materials with refugees registered with UNRWA's independent health system and holds their health records (10). Yet, there is a relative dearth of evidence about the effectiveness of mobile phone-based medical reminder platforms in refugee populations. Refugees face unique barriers to care: much of the Syrian population lives outside of formal refugee camps, in the slums of urban centers in search of economic opportunities (13). It is therefore hard to track refugee populations, maintain their health records, and ensure their awareness of the available services. The demand side of the problem is further aggravated by competing priorities (e.g., finding a job, schooling for children, etc.) for survival as a recent refugee. Despite these factors and the increasing usage of mobile phone- based reminders in low and middle-income countries (LMICs), few studies have examined refugee end-user attitudes toward these programs.

HERA, or Health Recording App, is a mobile health (mHealth) medical reminder intervention designed for the Syrian refugee population. It aims to leverage the high levels of smartphone usage in the community by providing specific and integrable solutions. HERA can be used for novel data collection, health information dissemination, and targeted health behavior changes for refugee populations. Furthermore, the HERA application enables users to receive healthcare appointment reminders, receive health information, centralize medical records, contact emergency services, and navigate the Turkish healthcare system in multiple languages.

A prior proof-of-concept study of this mHealth innovation was conducted in Turkey under the approval of an ethics review board in 2018, which informed the prototype of the mobile application and its initial features (14). This study aims to: (1) define the needs of Syrian refugee end-users, including specific barriers to healthcare access, (2) understand their perceptions on utility of a mobile health application for improving their healthcare-seeking behavior for antenatal care and vaccinations, and (3) enhance the user experience of the HERA mobile application by including end-users in the ideate mode of the application's design.

## Methods

### Context

The study site was located in Istanbul, Turkey, where almost one million Syrian refugees live. Study participants included 14 Syrian women who either had at least one child under the age of 2 years old or was pregnant at the time of recruitment. Participants primarily included individuals who had not attended a previous training session from the application developers on using the mobile application. The data collection period was conducted from July to November of 2019.

### Study design

The study used a grounded theory method with purposive sampling. The researchers conducted semi-structured and in-depth interviews of the participants using an exploratory approach to capture the individual experiences of using the application on the mobile device. The framework of the refugee interviews began by inquiring into their demographic background and the type of features they thought would be useful in an application for seeking preventive care, specifically prenatal care and vaccination services. This was followed with a direct observational component where the interviewer observed the interviewee navigate through the application to complete a given task. The interviewee was asked to narrate their thought process as they completed the task. Following the completion of the task, the participants shared their reflections on the mobile application.

A purposive sampling method was used to recruit at least nine participants that met the study criteria, at which, code definitions reach saturation or are typically found to stabilize (15–17). To increase generalizability, multiple regions in Istanbul where Syrians are known to reside in high numbers were targeted. To enroll participants, the research team contacted non-profit organizations that work with the Syrian population and clinics for Syrian patients. These organizations referred participants based on the refugee's availability and interest in participating. All those asked to be interviewed participated in the study. As refugees may be displaced or migrate, the interview was conducted in a single session. Participants were asked to download the application from an online application store (e.g., Google Play Store) during the interview if they had not already installed it prior to the training session.

The research protocol was developed by Turkish and American researchers who received graduate-level research training. The gender of the researchers was balanced and the field staff included a local female Turkish qualitative researcher and female Arabic interpreters of Turkish and Syrian origin. All field staff participated in a qualitative training process before piloting. The interview guide was first piloted with the help of the Arabic interpreters and was adjusted based on their feedback. Two pilot interviews were done to evaluate the external validity as well as translations of the questions, and the research team's efficiency in interviews.

Interviews were conducted by a research assistant and a translator, fluent in Arabic and Turkish, in either a meeting space provided by a non-profit organization or at the participant's house. Each interview was 1-h long. All interviews were conducted in privacy, with only the participant, the female interpreter, and interviewer. All interviews were audio recorded, following informed written and verbal consent. Audio recordings were directly transcribed verbatim in either Turkish or Arabic and subsequently translated to English by a native speaker. Interviews conducted with the assistance of an Arabic translator were also transcribed in Arabic and Turkish prior to English translation.

Open-ended individual interview guides were administered to pregnant and non-pregnant participants. The interview guide for non-pregnant participants focused on the child vaccination features and experiences, while the pregnant participants' guide focused on exploring their perspectives on prenatal health experiences and the mobile application features.

### Thematic coding and analysis

Three investigators (CM, AS, NN) reviewed the translated interview transcripts and wrote memos to capture prominent ideas. Key issues, concepts, and themes that emerged from the data were examined using constant comparison: each item was checked or compared with the rest of the data to identify and index analytical categories, or codes (17). All the developed codes were documented in a codebook. Summary reports of each code were created with examples of supporting text and reference to aid in the process of synthesizing data into key themes. Microsoft Excel 16.45 was used to manage the data (18). NVivo 12 was used to facilitate inductive data analysis and create diagrams (19).

### Ethical considerations

Institutional review board approval was obtained from Acibadem Mehmet Ali Aydinlar University Medical Research Ethical Board with decree number 2019-14/45 on 09/12/2019. An informed consent form in Turkish, English, and Arabic was prepared. The consent form participants were asked to sign was read and explained in Arabic by the interviewer. All but one participant agreed to be audio-recorded and everyone consented to participate.

## Results

### Participant demographics

A total of 14 participants were interviewed. Five were pregnant at the time of the interview, while the rest were not pregnant but had children. The median age of participants was 24 years (min = 19, max = 37). A majority of the participants had completed their lower secondary education (median years of education = 9). Other than two of the pregnant participants, all had at least one child at the time of the interview. Half of the participants had no fewer than two children (median = 2), and all but one did not have employment. The household had a median of 5 people (min = 3, max = 12) and a residing period of 4 years in Turkey. While one participant had arrived in Turkey only 3 months prior to the interview, the longest time spent in Turkey was 8 years. Additionally, three subjects had previously miscarried. A summary of individual participant demographics is presented in Table 1.

**Table 1:** Demographic characteristics of participants.

**Participant pseudonym**	**Age**	**Number of children**	**Highest level of education attained**	**Employment experience**	**Currently pregnant**	**Pregnancy month**	**Household size**	**Time in Turkey (years)**
*Aaliyah*	26	2	Primary education	Hairdresser	Yes	6	4	6
*Amara*	29	4	Lower secondary education	No	Yes	7	5	1
*Amina*	25	1	Lower secondary education	No	Yes	9	3	8
*Aisha*	19	0	Lower secondary education	No	Yes	2	4	4
*Amal*	24	0	Upper secondary education	No	Yes	8	4	4
*Calla*	24	2	Upper secondary education	No	No	-	4	6
*Cyra*	27	2	Lower secondary education	No	No	-	4	4
*Celina*	39	5	Lower secondary education	No	No	-	7	4
*Dani*	23	3	Lower secondary education	No	No	-	5	5
*Ezra*	24	3	Lower secondary education	No	No	-	5	4
Farrah^*^	24	1	Lower secondary education	No	No	-	2	0.25 (3 months)
Fatima^*^	30	2	Primary education	No	No	-	3	4
*Gul*	24	2	Lower secondary education	No	No	-	12	3
*Hyat*	36	6	Post- secondary vocational education	No	No	-	8	2

* Indicates the participant previously attended a HERA mobile application training session.

### Emergent themes

**Figure 1:**
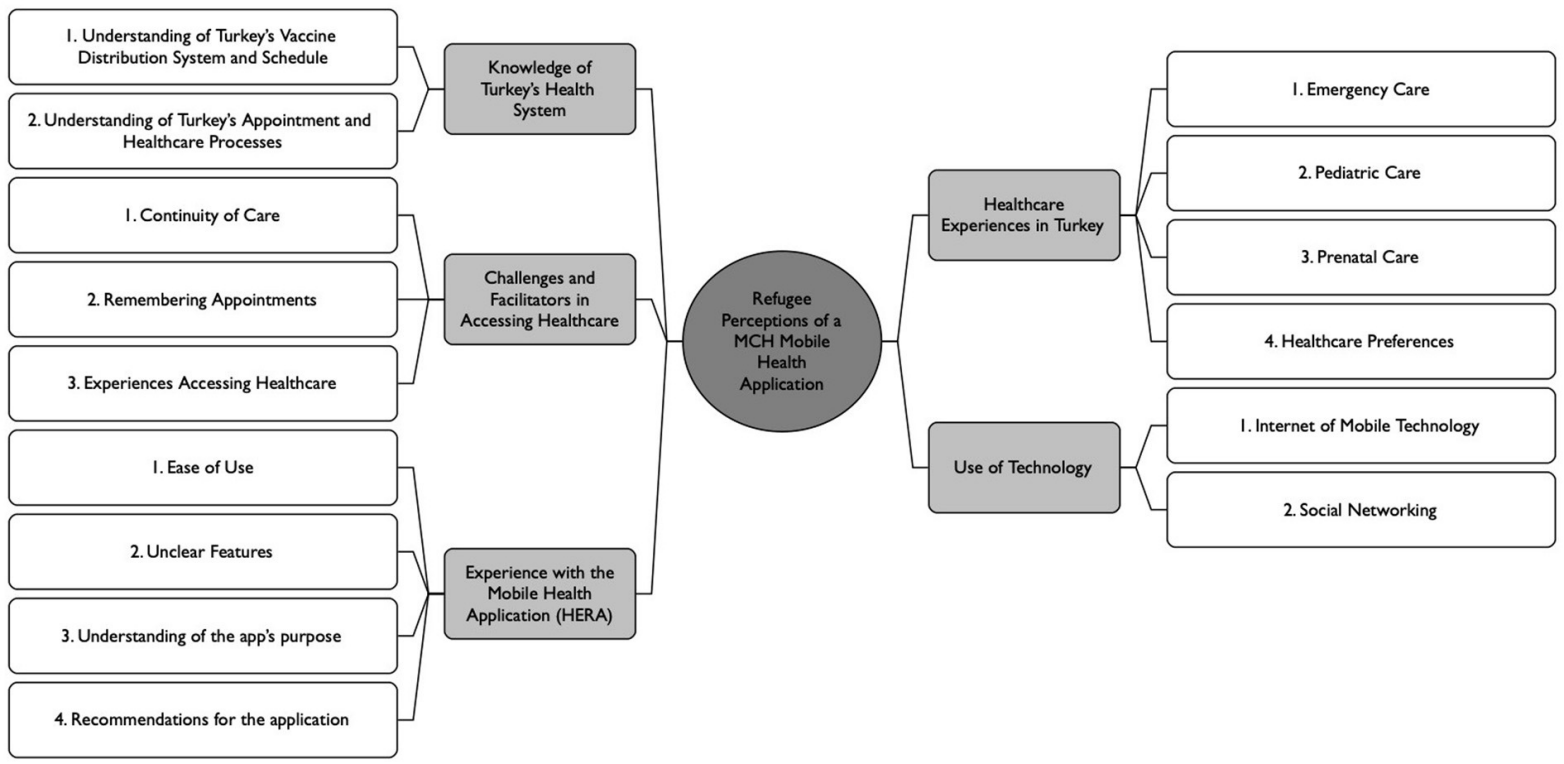
Coding tree diagram with themes and sub-themes.

Five overall emergent themes were identified: (1) Healthcare experiences in Turkey, (2) Knowledge of Turkey's healthcare system, (3) Challenges and facilitators of healthcare access, (4) Use of technology, and (5) Experience with the mHealth application. A coding tree diagram of the themes and sub-themes that emerged from the participant's responses is depicted in Figure 1.

### Healthcare experiences in Turkey

All participants reported prior experience with the healthcare system and hospital-level facilities in Turkey, either for themselves or their children. The choice of facility varied between private hospitals, public hospitals, and Syrian-run clinics. Emergency services were not frequently used as only two participants had previously utilized these services. One participant used the emergency services at a public hospital during her pregnancy because she had difficulties scheduling appointments at the public hospital and could not afford a private hospital (Aaliyah, 26).

Pregnant participants were asked about their healthcare experiences. Although Turkey offers free routine pregnancy services universally, none of the pregnant participants met the minimum clinically recommended number (*n* = 4) and frequency of prenatal clinic visits (20). Additionally, none of the participants had prepared a plan for their pregnancy due date, including selecting a health facility for delivery. While some of the participants had given birth in Syria or in other countries, none reported any difference in healthcare services they received during that time when compared with Turkey.

No participant reported any issues with their current pregnancy at the time of the interview. However, the subjects mentioned that the private hospitals in Turkey were generally more expensive and thus difficult to access.

“*I was going to a private hospital, but it was kind of expensive and my situation is difficult.” (Aaliyah, 26)*

The public polyclinics were reported to infrequently have specialized obstetrics and gynecological departments which led to referrals to more expensive private hospitals or physicians for services such as ultrasounds. The long wait time for public facilities also incentivized participants to seek care from private facilities. Wait times were also exacerbated if health records were not readily accessible for either the patient or the care provider at the point-of-care site.

“*At my last appointment, I had a problem. I forgot my health records at home, but I went there, and I was waiting for a couple of hours...they sent me back home to get the paper and come back. The time I spent in line went to waste and I had to come back again.” (Gul, 24)*

At least half of the participants said that they had received healthcare services from a non-Turkish provider. When study participants visited Syrian polyclinics, they typically used vaccination services and were referred to either private hospitals or Turkish physicians for gynecological services and other specialty care. The private hospitals could have Turkish doctors or doctors with different nationalities. One participant noted that additional healthcare services for Arabic speaking patients has been set up, which employs Arabic-speaking physicians (e.g., Syrian, Iraqi, and Palestinian trained-physicians). This service now has the capacity to provide prescriptions whereas previous Syrian polyclinics could not provide pharmaceutical services.

The children of participants generally received vaccinations, which could be administered at polyclinics. Only one participant indicated hesitancy toward the vaccination schedule, wanting to wait until her child was slightly older. Very few women reported already utilizing emergency services for severe diseases, accidents (e.g., drowning) or injuries for their children during their time in Turkey (*n* = 2). The personal health issues that some subjects experienced included perinatal infections with fever, chronic illness such as asthma, and mental health deterioration, including postpartum depression. Three subjects had experienced miscarriages, but this occurred prior to their arrival in Turkey. Health services that some subjects listed as important included more advanced prenatal care technology and mental healthcare resources as the Syrian female refugee population was often isolated during the pregnancy process.

“*...Most Syrian women are suffering from depression because they usually give birth alone, they do not have their mothers with them, they do not have their sisters with them. They feel alone, they feel tired of their children and their husbands. I felt very depressed after I gave birth.” (Hyat, 36)*

### Knowledge of Turkey's health system

Participants were asked about their knowledge of Turkey's health system and the process for accessing care. While participants were aware of the ambulance system, most did not know the contact number, or that it was free-of-charge for everyone in the country, including refugees and immigrants. It was also unclear to the participants what healthcare services were provided (7). None of the participants could comment on if Syria and Turkey had the same vaccination schedule.

Additionally, there was no standard procedure that the refugees followed to make healthcare appointments in Turkey. Some sought help from Turkish nationals to help them schedule appointments and navigate the healthcare system; while the others used the Central Physician Appointment System (Merkezi Hekim Randevu Sistemi, MHRS), a national unified call center to make appointments at second and third-tiered hospitals having translation services to assist non-Turkish people. Some participants also made appointments with private providers who had translators. One subject reported that she had attempted to download the MHRS mobile application but eventually used the MHRS call center because she did not have the government identification number necessary to use the application. Another subject scheduled the appointment through the local municipal government offices.

### Challenges and facilitators in accessing healthcare

#### Challenges

In terms of the participants' continuity of care, routine vaccinations were reported to be more regularly obtained compared to prenatal care. Migrant clinics (rather than hospitals) in Istanbul were the primary providers of pediatric care and vaccine services for the study subjects' vaccine-eligible children. Of the vaccine subjects (*n* = 8), only one subject reported that one of her children was unvaccinated because of the Syrian Civil War. However, the same subject's youngest child received all their vaccinations in Turkey (Fatima, 30). While some participants reported regularly getting prenatal care during their pregnancy, those who were not pregnant at the time of the interview reported receiving routine care less frequently.

Participants discussed several challenges when they accessed health services, including language, wait times at public facilities, expenses at private facilities, and uncertainty about the additional cost-free health services available to them as refugees, especially among those without Turkish identification documents. According to the participants, disparities in access to timely care was due to the Turkish bias toward Syrian refugees. Furthermore, as highlighted by a participant, the lack of transportation and childcare support exacerbated their difficulties with healthcare access.

Language was described as a considerable challenge by the study participants. Many indicated that their Turkish neighbors helped them set up appointments, or they preferred going to Syrian-run clinics.

“*I understand Turkish, but not much. Then, the Syrian doctors understand Turkish, but not much. I mean, for example, what does the doctor say? If I didn't understand something... then the Syrian doctor is better for me then” (Amal, 24)*

Wait times were often mentioned as an ongoing challenge in accessing healthcare from public healthcare facilities. For example, while one subject did not have a preference of where she wanted to give birth, another favored private over public facilities.

“*No thank God. I go to private hospitals–I don't go to public hospitals.” (Calla, 24)*

“*Only private hospitals, you never went to a public hospital?” (Interviewer)*

“*I went once and saw that it was really crowded so I only went to private hospitals.” (Calla, 24)*

The participants also faced long wait times at clinics for walk-in vaccination appointments.

“*One time [the doctor] told me to come back at nine and I went at ten, so I had to wait a while...about an hour and a half.” (Hyat, 36)*

According to the participants, their Syrian identity prevented them from accessing timely healthcare. A pregnant subject noted she had difficulties vaccinating her oldest child because one of the clinics she visited appeared not open to Syrians. Another participant felt that the long wait time at the public facility was a direct form of discrimination against Syrians.

“*Yes, I went there [a public hospital] but they made me wait so long [that] my child got so much worse, so I had to take her to a private hospital...I paid 300 liras and all of it was because we are Syrian.” (Cyra, 27)*

Besides the challenges mentioned above, the refugee participants had little to no knowledge of the healthcare benefits available to them. For example, one participant wanted to know if she needed an identification document for vaccinations (Farrah, 24). Another participant changed facilities because of unclear hospital requirements, such as whether government hospitals were allowed to charge money for prenatal services (Amina, 25).

Cost was an additional barrier. When asked about whether she received regular access to healthcare during current and past pregnancies, one participant who was 6 months into her current pregnancy, responded:

“*With my first son, yes, I used to go regularly but now I am not going because I do not have the money to go to a private doctor and I cannot make an appointment at a public hospital.” (Aaliyah, 26)*

The process of scheduling appointments was also difficult for the participants, especially during the season of Ramadan. As per the account of a study subject, appointments for routine prenatal care may also be scarce due to her difficulty in setting prenatal care appointments.

“*There is no [physical] line [at the public hospital]...Every time that I call them they postpone me to the next month then I come back again the next month and they tell me to come back the month after that so it has been six months like that...I went once to a private doctor on the European side and I called again but they told me there was no appointment” (Aaliyah, 26)*

Overall, participants did not find it difficult to remember scheduled medical appointments. The subjects discussed a variety of methods for remembering their medical appointments; some of the subjects who had to remember follow-up vaccine appointments referred to vaccination cards provided by the vaccinating facility with a designated follow-up date. One subject stated that the facility would call her if she missed her appointment, and another participant was reminded by her Turkish neighbor. Alternatively, study participants relied solely on their memory of the provider's verbal instruction to return for a follow-up appointment. According to another subject, the MHRS (the Ministry of Health's Central Appointment System) mobile application was also not easily accessible. In the case of setting and remembering vaccination appointments, most study subjects indicated placing their faith in physicians to provide the date that a child should make vaccination appointments. Physicians typically recorded this information on vaccination cards that the parents could refer to. However, one subject in the current investigation was not given a follow-up date.

#### Facilitators

Among the study participants, accessing healthcare was facilitated by more inclusive health systems and community support. For example, one subject noted that Syrian physicians staffing newly opened immigrant health centers made her feel more comfortable.

“*When they opened the health centers for immigrants, we relaxed because of the Syrian doctors.” (Aaliyah, 26)*

Moreover, as many of the subjects did not speak Turkish, Arabic translators in healthcare settings were commonly cited by participants as helpful during their healthcare experiences. Syrian physicians who speak Arabic fluently were preferred. Outside of the health system, two participants stated that their Turkish neighbors helped them navigate the healthcare system by making appointments on their behalf.

### Use of technology

All the participants were actively using their mobile phones. Social media applications and communication applications (e.g., WhatsApp) were the most common reason for using the phone other than contacting their family globally. While not a standard approach for scheduling appointments, such communication apps may also facilitate appointment scheduling with private facilities. However, one refugee participant did not have regular access to wireless internet and did not utilize communication applications because she was restricted to using her phone credit. In addition, some participants knew they could arrange their health appointments through the MHRS phone number; however, none of the participants successfully utilized the MHRS application. Among other routine uses of social media, one participant used her network to find customers for her informal hairdresser job where she provided services at her home.

### Experience with the mobile health application (HERA App)

Overall, the subjects said they found the mobile application easy to use and clear with regards to design. One participant had previously attended a training session with the HERA team. However, despite attendance at the training, the subject did not use the application for its designated purpose (e.g., she did not enter her children's information to calculate the vaccination date reminders). Some other individuals said they thought the application was useful but noted that they did not understand how to use it and would need more time to learn about it.

“*Do you find it difficult to open the application?” (Interviewer)*

“*I can learn little by little.” (Hyat, 36)*

One major feature that the participants liked about the application was that everything was accessible in Arabic, which is seldom the case for most of the online information. Another application feature, namely, the location finder for the nearest healthcare facility, was found to be particularly useful, especially when taking children to the hospital. One participant said that she can directly show it to the taxi driver, eliminating the need for giving directions and improving communication.

The location feature of the HERA application was the most-favored by multiple participants. Upon opening the mobile application, the photo-taking feature was not as obvious to users. However, once the purpose of the feature was clarified (e.g., storing medical records digitally), the participants seemed more interested in the feature. While the participants mostly understood the features, the mHealth application's relevance and feature navigation was unclear to some.

All the subjects said they would recommend the application to their pregnant friends or those with children. Participants had differing opinions on which feature they found most useful, with the most common answers being the vaccination reminders and the health information resources. Some participants also reported feeling less stressed about missing the vaccination appointments as the application could send them reminders.

Some participants felt reading about pregnancy, healthy nutrition, and other topics helped with their anxiety and worry about their pregnancy, especially for first-time pregnant women.

“*I liked that you can read a lot of information about pregnancy especially because I do not take care of myself and during pregnancy I feel very depressed, I sometimes feel suicidal.” (Aaliyah, 26)*

Responses on the mobile application's user experience design generally focused on content recommendations. Pregnant study subjects recommended including information on symptoms associated with pregnancy, as well as health and wellbeing advice for infants and mothers during the postpartum period. For example, more details on nutrition for infants, newborn health indicators (e.g., recommended height and weight based on age), and potential health complications for mothers (e.g., thyroid issues, postpartum depression) were requested.

One of the major concerns was data security and whether the application shared personal data and sensitive information with anyone. For example, a participant highlighted that some refugees do not have formal refugee status or identification and that some potential application users may be married at the age of 16, which is legal in Syria but not in Turkey.

Another issue that came up was the lack of phone memory. Participants were afraid that their added information to the application would deplete their phone's storage capacity. Participants were also concerned whether their data (e.g., digitally stored health records) would be retrievable if something happened to the mobile health application.

“*Did you download the application on your phone after the [mHealth app] training?”*

*(Interviewer)*

“*I did but my phone memory card is very bad, everything got deleted even the photos.” (Farrah, 24)*

“*The app is very nice, but what if it was deleted? I have a little girl and she likes to play with my phone so if I downloaded it again, will I have to reenter all the personal information?” (Calla, 24)*

Another critique of the mobile application's functionality highlighted that it was disconnected from Turkey's larger hospital health system.

“*It would be better if this application was for a hospital, and we went regularly to that hospital and we would use it there. It would be nice because we would go regularly to that hospital.” (Calla, 24)*

While a majority of subjects did not specify any changes in the design of the application (n = 8), scheduling medical appointments was desired by a participant. Although this could indicate an overall appreciation for the current mobile application's design and usability, alternative reasons for the limited responses may be: (1) the subjects had limited time to use the mobile application, and (2) social desirability bias in which social norms discourage the subjects from directly critiquing the mobile application in front of interviewers associated with the mobile application's developers.

## Discussion

The current research investigation aimed to provide insight into the most significant mHealth features which should be considered when designing mobile health applications for refugee populations. Overall feedback from this limited sample of users was positive; refugee women found the mobile application for preventive maternal-child healthcare extremely useful and were willing to recommend it to their peers. Important insights about the refugee healthcare experience for Syrian women living under temporary protection in Turkey were also explored in the study, including prior healthcare experiences, knowledge of the system, challenges to accessing care, use of technology, and specific application-related features.

Moreover, there appeared to be a high usage of mobile phones with internet capability among the study participants. Although the young median age (24 years) of study participants might have contributed to higher levels of mobile device use, in general, the Syrian population that has taken refuge in Turkey is very young; 50% are under the age of 18. Given the rapid growth of the immigrant population, with more than 400,000 Syrian refugee children born in Turkey since 2011 (21), high smartphone penetration may be viably leveraged for future refugee-directed interventions. There further appeared to be desire for understanding vaccine and antenatal care importance, with only one participant indicating hesitancy toward the vaccination schedule.

A few specific themes offer opportunities for further study. First, participants valued the vaccination reminder and the health information features the most. This is consistent with prior systematic reviews and a recent meta-analysis demonstrating improved vaccination uptake with health education and mobile phone appointment reminders as a pre-recorded message, particularly a combination of voice and text message, in low- and middle-income countries (LMIC) (22–24). Unfortunately, although the potential for mhealth interventions to improve vaccination coverage appears clear, existing studies in LMICs and vulnerable populations are reported as low to moderate quality (25). The dearth of rigorous studies in countries or populations facing the greatest barriers to immunization impedes evidence-based practice implementation in these settings.

Second, the use of emergency services was an important theme highlighted in this study. The participants had limited knowledge of available emergency services and the type of services provided. Although the participants did not appear to frequently use emergency services, it is possible that there was a lack of awareness that these services exist. For example, the emergency calling feature within the application recurrently received positive feedback, as the concept of a centralized ambulance system or services was not available in pre-war Syria. Participants who had been in Turkey for less time did not know what to do in emergency situations, representing future areas for targeted education and intervention.

Third, the impact of language barriers on the ability of refugee women to access healthcare was another consistently referenced opportunity. Although Turkey and Syria are neighboring countries, they do not share the same language or alphabet. Even prior to interfacing directly with the healthcare system, participants indicated the need for network assistance from Turkish neighbors to set up appointments, with a preference for Syrian-run clinics. The need for translation services often creates an informal market for interpreters within proximity to hospitals, to approach Arabic-speaking patients and provide assistance for a fee. Current interpreter services in hospitals are limited through a Turkish Ministry of Health employee. There is no reliable formal access to in-person translators, which may create additional utility for mobile-based services and refugee-specific interventions. Additionally, the participants viewed the application's Arabic language setting as one of the most favorable features.

The challenges that the refugee participants faced when seeking healthcare were critical in informing how to enhance the usability of a MCH mobile application for refugees in the Turkish context. The subjects often discussed how their healthcare decision-making process was deeply influenced by the trade-offs between the use of public and private facilities. Refugee patients discussed uncertainty about their ability to access specific services or facilities based on their identities as Syrian refugees or their official refugee status. While the Turkish health system has created a formal legal infrastructure for refugees to theoretically access care, discrimination continues to exist as the practice of administering such care is left to the discretion of individual providers. Systemic discrimination against Syrian refugees may also arise from the design of the health system. For example, provider payment incentives based on performance indicators (e.g., patient retention during routine prenatal follow-up appointments) can discourage providers from accepting migratory patient populations, including refugees, who may not as reliably follow up for care.

An additional challenge participants met was scheduling medical appointments. While some participants who were more familiar with the local resources were able to schedule appointments through Turkey's Central Physician Appointment System (MHRS), others would wait for an appointment at the facility. Some leveraged their extended social network by asking their Turkish neighbors to make appointments on their behalf. Since scheduling appointments can reduce appointment wait times and potentially increase the utilization of antenatal care (24), expanding appointment scheduling features or resources in the application may be beneficial for improving maternal-child healthcare.

The participants' recommendations for improving the mobile health application focused on expanding the available health information content, especially including resources on treating postpartum depression and mental health during perinatal care. The aspects of the mobile application the participants were most hesitant about centered around privacy concerns and data security, with regards to whether data was retrievable if a mobile device is replaced. Providing a clear explanation about the application's security protocols to ensure the privacy of their data, as well as providing secure cloud storage may improve refugee users' trust in the application's performance.

Previous research has cited “price value” as the cognitive trade-off between the monetary cost needed to use the application and the perceived quality or benefits from the application as a critical feature to increase the acceptance and use of mHealth technology (26). Although the HERA mhealth application is free for refugee users, access to affordable wireless internet and phone data may have implications on the application's perceived price value. The continued development of offline use could help mitigate this factor.

**Figure 2:**
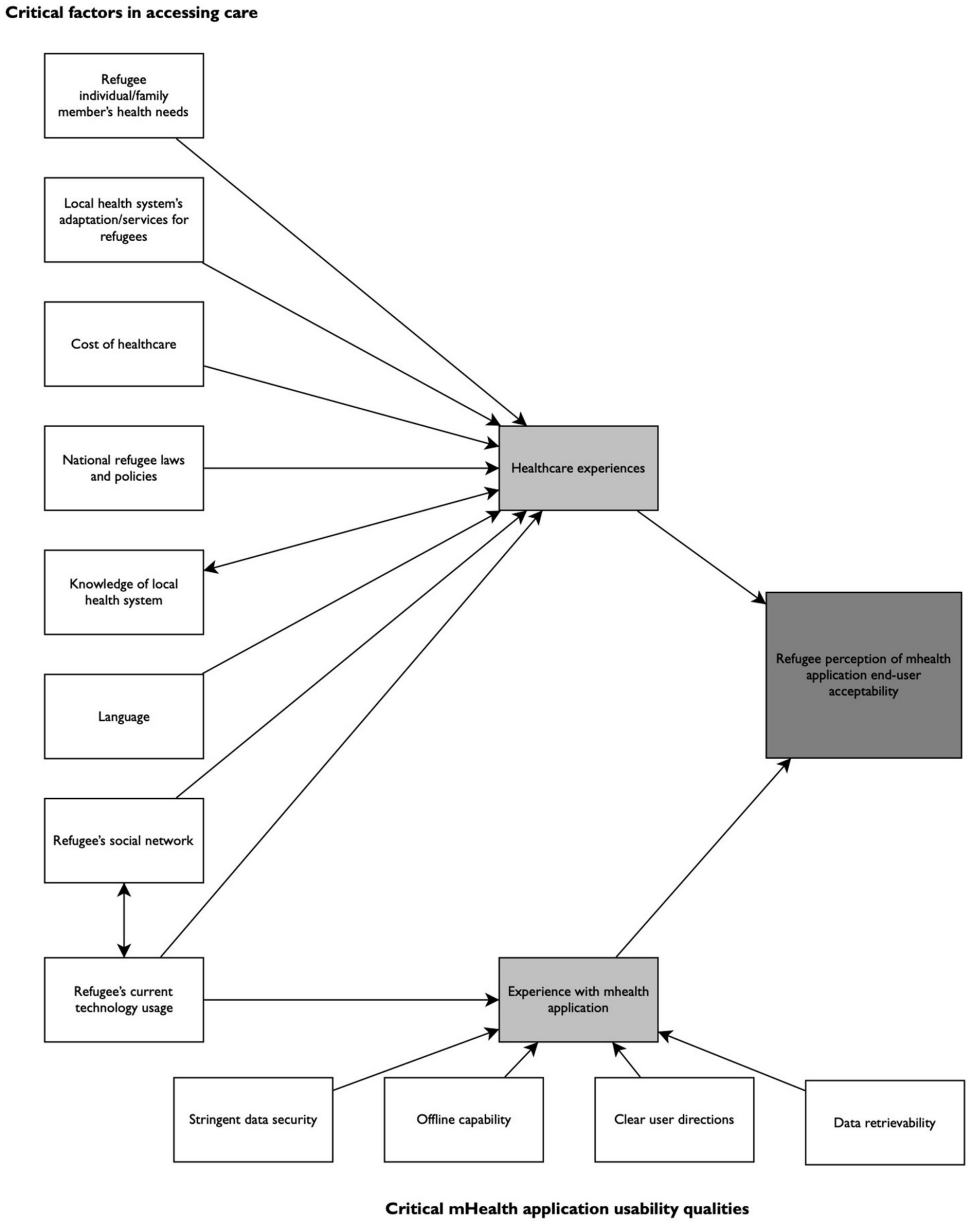
Considerations needed to enhance end-user acceptability of MCH-specific mHealth applications for refugees in Turkey.

Through an exploratory examination of the end-user perceptions of a novel mobile maternal-child healthcare application developed for Syrian refugee women, we were able to identify valuable considerations that refugee mobile health application developers should take to increase an application's acceptability. As depicted in Figure 2, a refugee's perception of a mobile health application is likely informed by their own healthcare experiences and the usability of the application. While there are many factors that influence an individual's healthcare experience, directly identified factors from Syrian refugee participants are critical for the ongoing content development of focused mHealth applications. Additionally, further exploration of the challenges and concerns the participants described with application use, can enhance uptake among this vulnerable patient population. The participants' concerns and questions about the described application inform critical mHealth usability qualities for current and future refugee mHealth applications.

### Strengths and limitations

Strengths of this study include a targeted focus on a doubly vulnerable population: Syrian refugee women is a study population often marginalized from standard data. The unique focus on the Turkish health system, which has been adapted to include refugees, provides a setting where financial access to healthcare does not introduce an additional barrier to care. Finally, local staff conducting this study using a grounded theory approach enabled the necessary rapport to allow for an exploratory understanding of users' actions in a local context, and with data depth and richness (27, 28).

The present study has several limitations. The research investigation relied on the participant's self-reported perceptions of the application, which, while important in exploratory studies, is susceptible to response and social desirability biases. The generalizability of the study is further limited by selection bias as the participants were recruited at local refugee organizations and the sample size is small. These participants may better reflect the experiences and interests of those with increased support as compared to more marginalized refugees at the periphery. Finally, sharing the application under facilitated supervision may have unintentionally provided application guidance for this subset of users, which may affect the generalizability of findings for future users in the community.

## Conclusion and future implications

This study explored perceptions, barriers, and facilitators to enhance the acceptability of a mobile application in refugee populations. User experiences have been categorized at the intersection of critical factors in accessing care and critical mHealth application qualities, to describe refugee healthcare experiences and their perceptions of a novel mHealth application. Based on the end-users' direct experience of the HERA mHealth application, several practical adaptations can be further explored. More basic and upfront education of the specific goals of the mHealth application and its personal significance to the user should be provided, prior to education about specific application features, in order to optimize buy-in and sustainability of use.

Finally, expanded integration within the health system for more bi-directional interface with hospitals and clinics may increase application utility if healthcare providers can also view and add specific visit-related information. The mobile application intervention's theory of change premise is to facilitate the user's understanding and navigation of the health system to regularly access care. As such, enhancing the application's inter-operability with the subjects' health system could be beneficial for attaining the desired user experience and the application's purpose. However, data security and privacy for a politically vulnerable population would remain an important challenge in the implementation of these adaptations.

Exploring the acceptability of similar types of mHealth solutions will require further research on the readiness of (1) local infrastructure to accommodate the integration of mHealth, and (2) national level stakeholder collaboration to ensure health systems integration. While this study provides promise into initial user perceptions, further prospective research is needed on long-term retention and use of mHealth applications for refugee women and other displaced populations.

## Data availability statement

The raw data supporting the conclusions of this article will be made available by the authors, without undue reservation.

## Ethics statement

The studies involving human participants were reviewed and approved by Acibadem Mehmet Ali Aydinlar University Medical Research Ethical Board. The patients/participants provided their written informed consent to participate in this study.

## Author contributions

CM and AS designed and implemented the intervention and coordinated all aspects of the study. CM performed data collection, data analysis, and drafted the initial manuscript results. AS and NN assisted with qualitative analysis and coding. AS drafted initial methods. NN drafted the discussion as well as provided critical manuscript revisions and advisory support. CH drafted the introduction and provided critical revisions of the entire manuscript. All authors agree on the final submitted version of the manuscript.

## Conflict of interest

Authors AS and NN are founders of HERA Digital Health, a non-profit that created HERA App, the described open source mhealth intervention. Neither receive funding or compensation for this role.

The remaining authors declare that the research was conducted in the absence of any commercial or financial relationships that could be construed as a potential conflict of interest.

## Publisher's note

All claims expressed in this article are solely those of the authors and do not necessarily represent those of their affiliated organizations, or those of the publisher, the editors and the reviewers. Any product that may be evaluated in this article, or claim that may be made by its manufacturer, is not guaranteed or endorsed by the publisher.
