# Supplementary material for: Perceptions on a mobile health intervention to improve maternal child health for Syrian refugees in Turkey: Opportunities and challenges for end-user acceptability
Source: Front Public Health. 2022 Nov 22;10:1025675. doi: 10.3389/fpubh.2022.1025675 (PMC9722941; doi:10.3389/fpubh.2022.1025675)
Supplement: Supplementary file 3 [file Table_3.DOCX]

**HERA User Experience Research Study Topic Guide (Pregnant Women)**

**Demographic Information:**

1. May you share your birth date?
2. Do you currently work?
3. When did you attend school until?
4. How many children have you given birth to?
   1. How many are currently alive?
5. How many years/months have you spent in Turkey?
6. How many people are in your household?

*Initial in-depth interview component*

**Domains: Mobile Device Usage; Healthcare Experience; User Preference**

***Mobile Device Usage***

1. If you have a mobile phone here in Turkey, can you please describe how you use it?
2. How have you used your phone to help you access healthcare, if at all?

***Healthcare Experience***

1. Thinking back to your last pregnancy, what are some of the biggest challenges to seeking care?

***User Preference***

*(During pilot, may want to switch around question order to see if responses differ)*

1. How could your phone better help you see the doctor for pregnancy-related healthcare, if at all?
2. What information or tool do you think would best help you visit the doctor for pregnancy-related healthcare, if any?

*Direct observational component*

[Directions for think-aloud]

1. Can you enter your basic health information about your pregnancy?
2. Can you find a clinic/hospital near where you live that you would go to for your pregnancy?
3. Can you upload a photo to the electronic health record feature?
   1. Please delete it.
4. How can you see when you should next visit your doctor for a checkup?

*Review in-depth interview component*

**Domains: Relevance of resources; Ease of use (intuitive); Challenges to using application**

Warm up:

1. Can you describe your initial feelings about the HERA application?

Ease of use:

1. What difficulties did you have completing the given tasks in the mobile application?
2. How would you improve the application to make it easier to use?

Relevance of resources:

1. Prompt: Can you describe what makes a mobile application useful?
2. What do you think of the usefulness of the applications different features?
3. Which feature(s) did you like the most?
   1. What aspects of those features made you feel this way?
4. Which feature(s) did you like the least?
   1. What aspects of those features made you feel this way?

*[Comment: The following is if we needed to make more targeted questions, but timing is an issue to consider]*

Reminders:

1. What do you think about the reminder feature?
   1. Probe: What do you like and/or dislike about the reminders?
   2. Probe: How could it best be improved to make you more likely to use it, if at all?

Health facility map feature:

1. Why is the map feature of the healthcare clinics useful or not useful?
2. Why would you go or not go to the clinic that you found on the map feature of the HERA application?
3. When seeking healthcare, have you seen a Syrian physician outside of a formal public Turkish hospital or clinic?
   1. Probe: Can you describe how this experience compares to the formal public Turkish hospitals/clinics?

Electronic Health Records:

1. What do you think about the electronic health record feature?
   1. Probe: What do you like and/or dislike about the reminders?
   2. Probe: How could it best be improved to make you more likely to use it, if at all?

Challenges to using the application:

1. What are some of the challenges you expect you may have using the mobile application, if any?

**Concluding:**

1. Does the HERA application make you feel more comfortable seeing a doctor in Turkey about your pregnancy, if at all?
2. Is there anything else you would like to share with us about the HERA mobile application?
3. Would you recommend this app to others?
   1. Who else would you recommend the app to?
